# Analysis of the characteristics of endometrial fluid samples in recurrent pregnancy loss patients based on 16S rRNA gene sequencing technology

**DOI:** 10.3389/fcimb.2025.1646125

**Published:** 2025-10-13

**Authors:** Xiaoping Wang, Nan Ding, Xiaofeng Li, Menghao Lu, Peili Wang

**Affiliations:** Reproductive Medicine Center, Lanzhou University Second Hospital, Lanzhou, China

**Keywords:** recurrent pregnancy lose, endometrial fluid, 16S rRNA, microbial dysbiosis, microbiota

## Abstract

**Introduction:**

Recurrent pregnancy loss (RPL) is a complex condition with multifactorial causes. This study aimed to characterize the microbial composition of endometrial fluid in RPL patients compared with healthy controls.

**Methods:**

A total of 114 participants were recruited from the Second Hospital of Lanzhou University between March and September 2023, including 68 RPL patients and 46 healthy controls. Clinical data and endometrial fluid samples were collected. Microbial DNA was extracted and analyzed using 16S rRNA gene sequencing. Microbial diversity was assessed with QIIME, semi-partial correlation analysis was applied to explore associations between groups, and LEfSe was used to identify differentially abundant taxa.

**Results:**

No significant differences in alpha diversity indices were found between the groups (p>0.05). However, beta diversity showed significant differences (p<0.05), indicating distinct microbial compositions. At the genus level, *Vibrio* and *Pseudoalteromonas* were positively correlated with RPL, suggesting their potential role in the condition. LEfSe analysis further validated our results, highlighting several taxa with significant differences in abundance, indicating microbial imbalances in RPL patients.

**Discussion:**

The study emphasizes the impact of microbial imbalances on reproductive health, potentially aiding in the development of targeted interventions to restore microbial balance and improve pregnancy outcomes in RPL patients.

## Introduction

Recurrent Pregnancy Loss (RPL) is one of the most challenging issues in reproductive medicine due to its complex etiology and the limited availability of effective treatments. The European Society of Human Reproduction and Embryology defines RPL as two or more consecutive spontaneous miscarriages, affecting 1-3% of couples attempting to conceive (Bender Atik et al., 2018; Zhao et al., 2022). The endometrium, as a crucial interface between the mother and the embryo, undergoes dynamic changes to facilitate the complex interactions necessary for embryo development (Pirtea et al., 2021). Any disruption in this process can lead to pregnancy loss, significantly impacting the physical and mental health of the mother (Benkhalifa et al., 2022). The causes of RPL are multifaceted, with potential etiological pathways being complex and involving multiple factors. Now, it is evident that the endometrial microenvironment plays a pivotal role in early embryo-endometrium interactions (Li et al., 2022; Odendaal et al., 2024).

The role of reproductive tract microbiota in the pathogenesis of gynecological infectious diseases, tumors, and endocrine disorders has become a focus of research in obstetrics and gynecology (Zhu et al., 2022). Numerous studies on lower reproductive tract microbiota have demonstrated that alterations in the vaginal microbiome are associated with adverse reproductive events, particularly the shift from Lactobacillus dominance to increased microbial diversity (Chen et al., 2021; Gao et al., 2024). However, there are relatively few studies on the association between endometrial microbiota and RPL. Although the endometrium has long been considered sterile (Perez-Muñoz et al., 2017), advances in technology and a deeper understanding of the uterine microenvironment have led to studies characterizing and quantifying the endometrial microbiota, suggesting a link between endometrial microbiota and RPL. Recent research has started to focus on the potential role of the endometrial microbiota in reproductive outcomes (Peuranpää et al., 2022; Takimoto et al., 2023; Odendaal et al., 2024).

In the context of RPL, understanding the role of the endometrial microbiota becomes particularly compelling. Our study aims to elucidate the microbial patterns in endometrial fluid samples from patients with RPL compared to those with normal controls, utilizing 16S rRNA gene sequencing technology. By doing so, we hope to shed light on the association between dysregulated endometrial microbiota and reproductive failure, providing new perspectives for the prevention of RPL.

## Material and methods

### Subjects and study design

We constructed the case-control study in Lanzhou University Second Hospital Reproductive Medicine Center from March 2023 to September 2023.This study was conducted in accordance with the principles outlined in the Declaration of Helsinki. The research protocol was approved by the Ethics Committee of the Second Hospital of Lanzhou University (Approval Number: 2022A-430). All participants provided written informed consent before participation in the study. No identifiable personal information is presented in the manuscript, and all data have been anonymized to ensure confidentiality. A total of 114 women were included in this study. The study group consisted of 68 patients with RPL, and 46 healthy individuals served as the control group. All participants in both the RPL and control groups conceived naturally. The inclusion criteria were as follows: (1) women of reproductive age; (2) no sexual activity, vaginal procedures, or douching within the past 48 hours; (3) no use of systemic or local vaginal antibiotics or probiotics within the past 30 days; (4) no urogenital inflammation or other body infections within the past 3 months; (5) all participants provided written informed consent. The exclusion criteria included: (1) anatomical abnormalities, chromosomal abnormalities, thrombophilic conditions, antiphospholipid syndrome, and endocrine diseases; (2) significant organic diseases such as hypertension, immune system diseases, and tumors, as well as severe mental or psychological disorders. For the RPL group, participants were required to have experienced two or more consecutive pregnancy losses within the past year, with all losses occurring before the 24th week of gestation. Healthy controls were defined as women who had successfully carried at least one pregnancy to term without a history of pregnancy loss.

### Clinical data collection and microbial sampling procedure

Medical records were meticulously reviewed, and the following data were assessed: maternal demographic characteristics such as age, education, race, and body mass index (BMI). Additionally, fasting blood glucose (FBG) and lipid metabolism variables including total cholesterol (TC), triglycerides (TG), high-density lipoprotein (HDL), and low-density lipoprotein (LDL) were considered.

The microbial sample collection process followed the 2014 Standard Operating Procedure (SOP) and the endometrial fluid collection guidelines established by the World Endometriosis Society to ensure methodological rigor and reproducibility (Lin et al., 2024). Specifically, endometrial fluid samples were collected between days 2–7 of the menstrual cycle from participants meeting the inclusion and exclusion criteria, provided they had no abnormal vaginal secretions upon examination. Patients were placed in the lithotomy position, and thorough aseptic precautions were undertaken: the vulva and vagina were disinfected three times with povidone-iodine solution, followed by cervical and external os disinfection. Additionally, a sterile cotton swab was used to cleanse the cervical os three times to minimize contamination. A sterile AIC18 insemination catheter was then gently introduced into the uterine cavity. After removing the inner tube, 5 mL of sterile saline was instilled into the cavity and immediately aspirated. The collected lavage fluid was transferred into a pre-cooled sterile cryotube containing 2 mL of sterile saline, which had been pre-frozen at -80 °C. The samples were then promptly stored at -80 °C until further analysis.

### 16S rRNA sequencing analysis

#### Extraction of genomic DNA

Microbial genomic DNA was extracted using the cetyltrimethylammonium bromide (CTAB) method, following a standardized protocol. Briefly, 1000 μL of CTAB lysis buffer was added into a 2.0 mL microcentrifuge tube, and lysozyme was added before transferring the sample. The mixture was incubated at 65 °C with intermittent inversion to ensure sufficient lysis. After centrifugation, the supernatant was sequentially extracted with phenol:chloroform:isoamyl alcohol (25:24:1, pH 8.0) and chloroform:isoamyl alcohol (24:1), each followed by centrifugation at 12,000 rpm for 10 min. DNA was precipitated with isopropanol at –20 °C, collected by centrifugation at 12,000 rpm for 10 min, and washed twice with 1 mL of 75% ethanol. The pellet was air-dried under sterile conditions (avoiding overdrying) and dissolved in ddH_2_O, with optional incubation at 55–60 °C for 10 min to facilitate solubilization. Residual RNA was digested by adding 1 μL of RNase A and incubating at 37 °C for 15 min.

#### Amplicon generation

Distinct regions of the 16S rRNA, 18S rRNA, and ITS genes were amplified using specific primers with barcodes (e.g., 16SV4: 515F-806R, 18SV4: 528F-706R, 18SV9: 1380F-1510R). The PCR reaction mixture consisted of 15 µL Phusion^®^ High-Fidelity PCR Master Mix (New England Biolabs), 0.2 µM of each primer, and about 10 ng of template DNA. The thermal cycling conditions were: initial denaturation at 98°C for 1 minute, followed by 30 cycles of 98°C for 10 seconds, 50°C for 30 seconds, and 72°C for 30 seconds, with a final extension at 72°C for 5 minutes.

#### PCR products quantification and qualification

The PCR products were purified using magnetic bead purification. Samples were then mixed in equal density ratios based on the concentration of the PCR products. After thorough mixing, the target bands were extracted. Negative controls consisting of nuclease-free water from the commercial kit were included during the PCR amplification stage, processed in parallel with clinical samples, and sequenced to monitor contamination (Bokulich et al., 2013). These negative controls yielded either no reads or only negligible reads (<10), which were considered background noise rather than true microbial signals. Accordingly, no valid microbial reads were detected in the negative controls, supporting the reliability of the sequencing results. All experimental procedures were performed under sterile conditions to minimize the risk of contamination.

### Library preparation and sequencing

Sequencing libraries were prepared and indexed. The libraries were then quantified using Qubit and real-time PCR, and their size distribution was verified with a bioanalyzer. Once quantified, the libraries were pooled and sequenced on Illumina platforms according to the effective library concentration and required data amount.

### Paired-end reads assembly and quality control

Paired-end reads were assigned to samples based on their unique barcodes and truncated to remove the barcode and primer sequences. The paired-end reads were then merged using FLASH (V1.2.11) and quality-filtered with fastp (Version 0.23.1) to obtain high-quality clean tags. Chimera sequences were detected and eliminated using the vsearch package (V2.16.0) to produce effective tags.

### ASVs denoise and species annotation

Denoising was carried out using the DADA2 module within QIIME2 (Version QIIME2-202202) to generate initial Amplicon Sequence Variants (ASVs). Species annotation was subsequently performed using QIIME2 with the Silva database for 16S rRNA gene sequences.

### Alpha diversity and beta diversity

Alpha diversity indices, including Observed features, Chao1, Simpson, and Shannon, were computed using QIIME2 to assess the diversity, richness, and uniformity of microbial communities in the samples. Beta diversity was evaluated based on weighted UniFrac distances in QIIME2 to determine the complexity of community composition and compare differences among samples or groups. A heatmap of UniFrac distances was created, and cluster analysis was conducted using Principal Coordinate Analysis (PCoA) in R software (Version 4.3.1) with the ade4 and ggplot2 packages.

### Statistical analysis

Clinical sample data were collected and analyzed. Continuous variables are presented as mean ± standard deviation (SD) or median (25th–75th percentiles), as appropriate. Group differences were analyzed using the t-test or the Kruskal-wallis rank sum test for continuous variables. Categorical variables are presented as frequencies, and the chi-square test was used to assess differences between groups. Alpha diversity indices included Observed features, Chao, Simpson and Shannon, which were used to analyze the diversity, richness, and uniformity of microbial communities in the samples. Beta diversity was assessed using UniFrac distances to study the structural variation of microbial communities among samples. Principal Coordinate Analysis (PCoA) was employed for visualization, which is a dimensionality reduction method based on distance matrices to find principal coordinates. To identify microbial taxa associated with RPL and adjust for potential confounding variables such as age, BMI, education, race, fasting blood glucose (FBG), and lipid profiles (total cholesterol, triglycerides, HDL, and LDL), Semi-partial Spearman correlation tests were performed using the R package pcor [code: pcor.test(data, group, c(age, BMI, education, race, FBG, TC, TG, HDL, LDL))]. This analysis allowed us to determine which microbial taxa were associated with RPL after adjusting for these confounders. Significant differences between the two groups were also identified using LEfSe (Linear Discriminant Analysis Effect Size), which detects biomarkers with statistically significant differences in abundance between groups. LEfSe combines statistical significance with biological relevance, providing an LDA (Linear Discriminant Analysis) score to indicate the effect size of each differentially abundant feature. The threshold for LEfSe analysis in this study was set to LDA >4 and P < 0.05.

## Results

### Patient characteristics

From March 2023 to September 2023, 114 women participated in this study: 46 in the control group and 68 in the RPL group. There was a significant difference in age between the groups, with the control group being older (p = 0.010). The control group also exhibited higher height (p = 0.002), weight (p = 0.011), and slightly higher BMI, though the latter was not statistically significant (p = 0.058). Educational attainment differed significantly, with a higher proportion of college-educated individuals in the RPL group (p < 0.001). The majority of participants were Han Chinese, with no significant difference in ethnicity distribution (p = 0.082). The control group had significantly higher LDL levels (p = 0.008), while other biochemical markers like FBG, TC, TG, and HDL did not show significant differences(**Table 1**).

**Table 1:** Patients baseline of clinical features analysis.

Variables	Total (n = 114)	Control (n = 46)	RPL (n = 68)	*p*-value
Age (year)	32 (30, 36.8)	34 (31.2, 37.8)	32 (29, 35)	0.010
Height (cm)	160 (158, 166)	165 (160, 170)	160 (158, 165)	0.002
weight (Kg)	59.2 (53.2, 65)	63 (54, 71.1)	57.2 (53, 63.2)	0.011
BMI (kg/m^2^)	23 ± 3.2	23.7 ± 3.6	22.5 ± 2.7	0.058
Education, n (%)				< 0.001
High school and below	48 (42.1)	35 (76.1)	13 (19.1)	
High school	15 (13.2)	7 (15.2)	8 (11.8)	
College and above	51 (44.7)	4 (8.7)	47 (69.1)	
Race, n (%)				0.082
Han Chinese	105 (92.1)	45 (97.8)	60 (88.2)	
Other ethnicities	9 (7.9)	1 (2.2)	8 (11.8)	
FBG (mmol/L)	5 (4.8, 5.3)	5 (4.8, 5.4)	5 (4.7, 5.2)	0.211
TC (mmol/L)	4.2 (3.7, 4.8)	4.4 (4, 4.8)	4.1 (3.5, 4.7)	0.089
TG (mmol/L)	1.2 (0.8, 1.5)	1.2 (0.9, 1.4)	1 (0.8, 1.6)	0.412
LDL (mmol/L)	2.7 (2.2, 3.1)	2.8 (2.6, 3.2)	2.6 (2.1, 3)	0.008
HDL (mmol/L)	1.3 (1.1, 1.4)	1.3 (1.1, 1.4)	1.4 (1.1, 1.5)	0.106

BMI, Body Mass Index; FBG, Fasting Blood Glucose; TC, Total Cholesterol; TG, Triglycerides; LDL, Low-Density Lipoprotein; HDL, High-Density Lipoprotein.

### Distribution of endometrial fluid microbiota in patients

#### Phylum-level analysis

**Figure 1:**
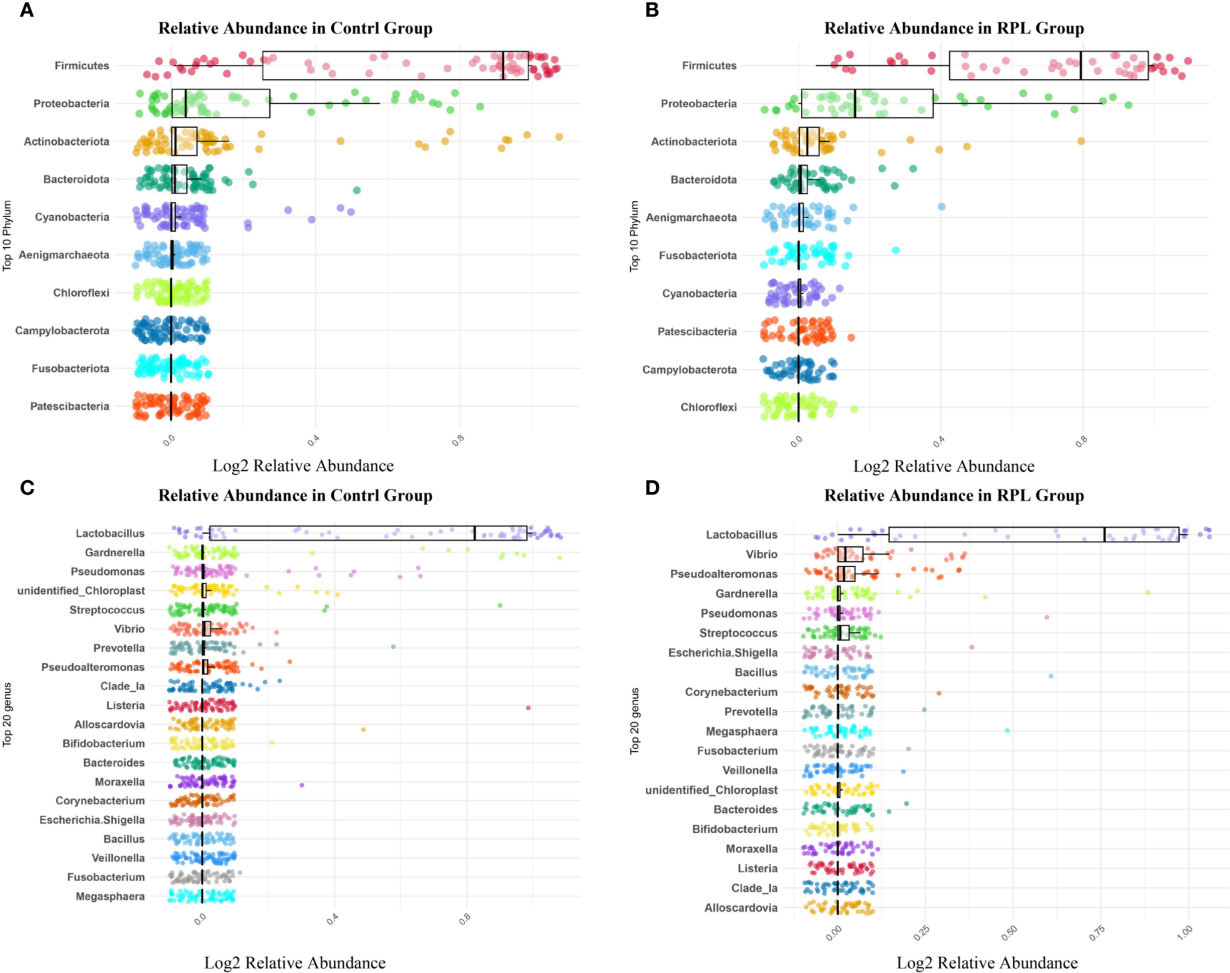
Microbial composition in the control group and the RPL group. **(A)** Relative abundance of the top 10 phyla in the control group. **(B)** Relative abundance of the top 10 phyla in the RPL group. **(C)** Relative abundance of the top 20 genera in the control group. **(D)** Relative abundance of the top 20 genera in the RPL group.

At the phylum level, the microbial composition shows notable differences between the control and RPL group. In the control group (**Figure 1A**), the most abundant phyla include *Firmicutes, Proteobacteria, Actinobacteriota*, and *Bacteroidota*, with *Firmicutes* being the most dominant. Other phyla present include *Cyanobacteria, Aenigmarchaeota, Chloroflexi, Campylobacterota, Fusobacteriota*, and *Patescibacteria*. In comparison, the RPL group (**Figure 1B**) showed a distinct shift in their microbial composition. While *Firmicutes, Proteobacteria, Actinobacteriota* and *Bacteroidota* remained the most abundant phyla. The relative abundance of other identified phyla included *Fusobacteriota, Cyanobacteria, Patescibacteria, Campylobacterota* and *Chloroflexi* showed variations compared to the control group.

#### Genus-level analysis

At the genus level, the control group (**Figure 1C**) illustrates the top 10 genera were *Lactobacillus, Gardnerella, Pseudomonas, Unidentified Chloroplast, Streptococcus, Vibrio, Prevotella, Pseudolateromonas, Clade_Ia*, and *Listeria*. *Lactobacillus* exhibited the highest relative abundance, indicating its dominant presence and its known role in maintaining a healthy endometrial environment. Other genera such as *Gardnerella, Pseudomonas*, and *Streptococcus* also showed notable abundance, while genera like *Vibrio* and *Prevotella* were present in lower amounts. In the RPL group (**Figure 1D**), the top 10 genera were *Lactobacillus, Vibrio, Pseudolateromonas, Gardnerella, Pseudomonas, Streptococcus, Escherichia.Shigella, Bacillus, Corynebacterium*, and *Prevotella*. Additionally, the RPL group showed higher levels of *Vibrio, Pseudolateromonas*, and *Bacillus*. These differences in the genus-level distribution highlight significant shifts in the microbial communities between the control and RPL group.

### Beta diversity and alpha diversity

**Figure 2:**
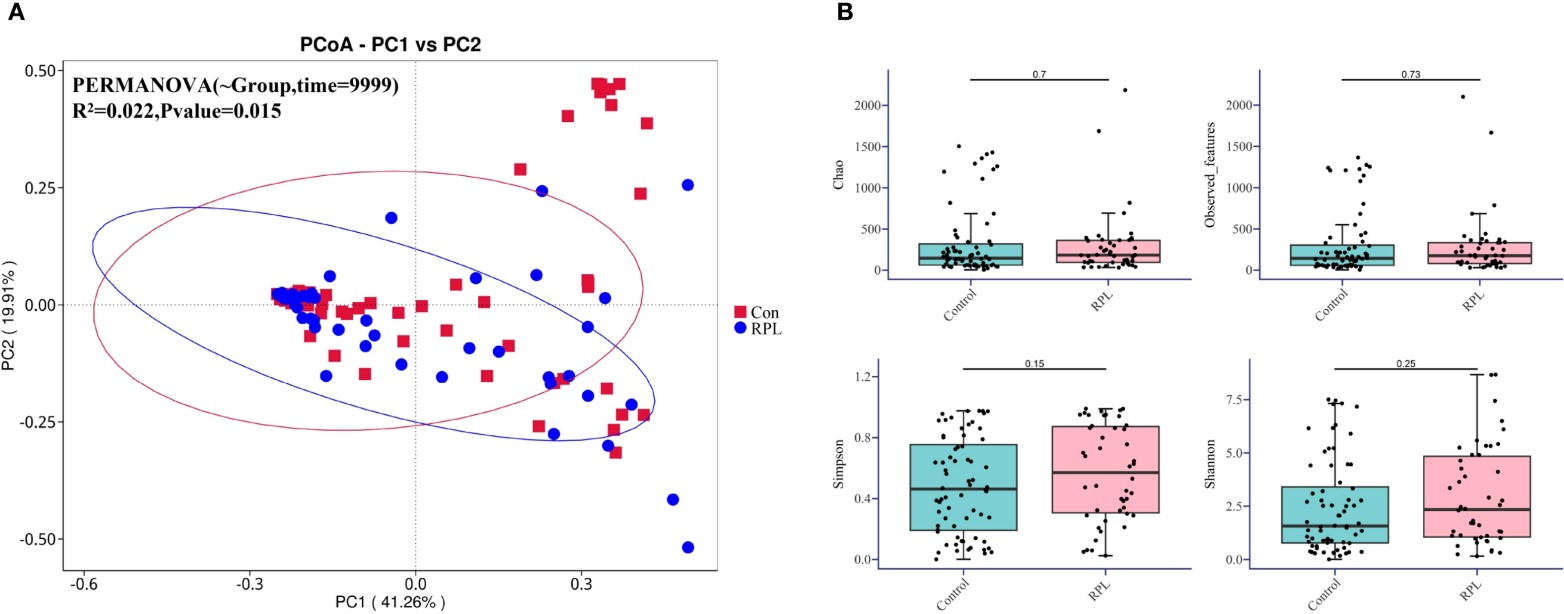
Microbial diversity in the control group and the RPL group. **(A)** Principal Coordinates Analysis (PCoA) plot based on β-diversity. The plot shows the separation of microbial communities between the control (red squares) and RPL (blue circles) group. **(B)** Box plots depicting α-diversity indices (Chao, Observed Features, Simpson, and Shannon) for the control and RPL group.

The β-diversity analysis illustrated by the Principal Coordinates Analysis (PCoA) plot in **Figure 2A** reveals a significant separation between the microbial communities of the control group (red squares) and the RPL group (blue circles). This separation suggests distinct microbial profiles between the two groups. PERMANOVA analysis confirms this difference with statistical significance (R²=0.022, p=0.015). In terms of α-diversity, as shown in **Figure 2B**, there are no statistically significant differences between the control and RPL group across various indices, including Chao1 (p=0.7), Observed Features (p=0.73), Simpson (p=0.15), and Shannon indices (p=0.25). These results indicate that there are no statistically significant differences in the two groups. However, beta diversity analysis showed significant differences, suggesting distinct microbial community structures in RPL patients compared to controls.

### Endometrial fluid relative abundance composition and correlation in patients with RPL

**Figure 3:**
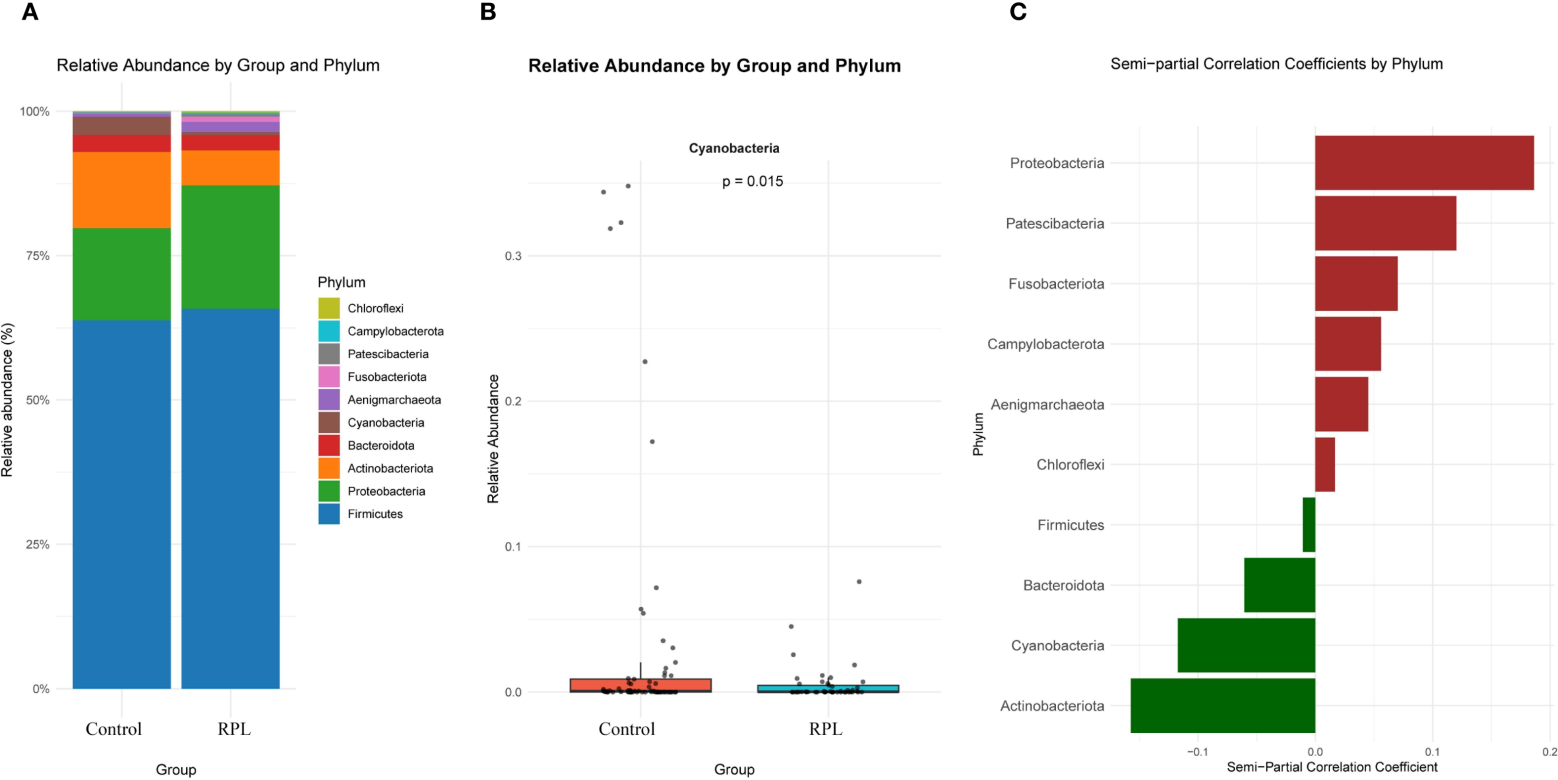
Microbial composition and semi-partial correlation analysis at the phylum level between the control group and the RPL group.**(A)** Relative abundance of the top 10 phyla in the control and RPL group. **(B)** Relative abundance of Cyanobacteria, showing a significant difference between the control and RPL group (p=0.015). **(C)** Semi-partial correlation coefficients of different phyla with RPL after adjusting for clinical variables (age, BMI, education level, race, FBG, TC, TG, LDL, and HDL). Positive correlations with RPL are shown in red, and negative correlations are shown in green.

At the phylum level, the relative abundance of various phyla between the control and RPL group is depicted in **Figure 3A**. The most abundant phyla include *Firmicutes, Bacteroidota*, and *Proteobacteria*. **Figure 3B** presents the relative abundance of phyla that show significant differences between the two groups. Notably, *Cyanobacteria* is more abundant in the control group compared to the RPL group. **Figure 3C** highlights the phyla that are positively and negatively correlated with RPL after adjusting for clinical variables (including age, BMI, education level, race, FBG, TC, TG, LDL, and HDL) using semi-partial correlation analysis. *Bacteroidota* and *Cyanobacteria* are positively correlated with RPL. however, *Cyanobacteria* does not show a statistically significant difference between the two groups after adjusting for confounding factors. The results of the semi-partial correlation analysis at the phylum level are presented in **Supplementary Data** – **Supplementary Table S1**.

**Figure 4:**
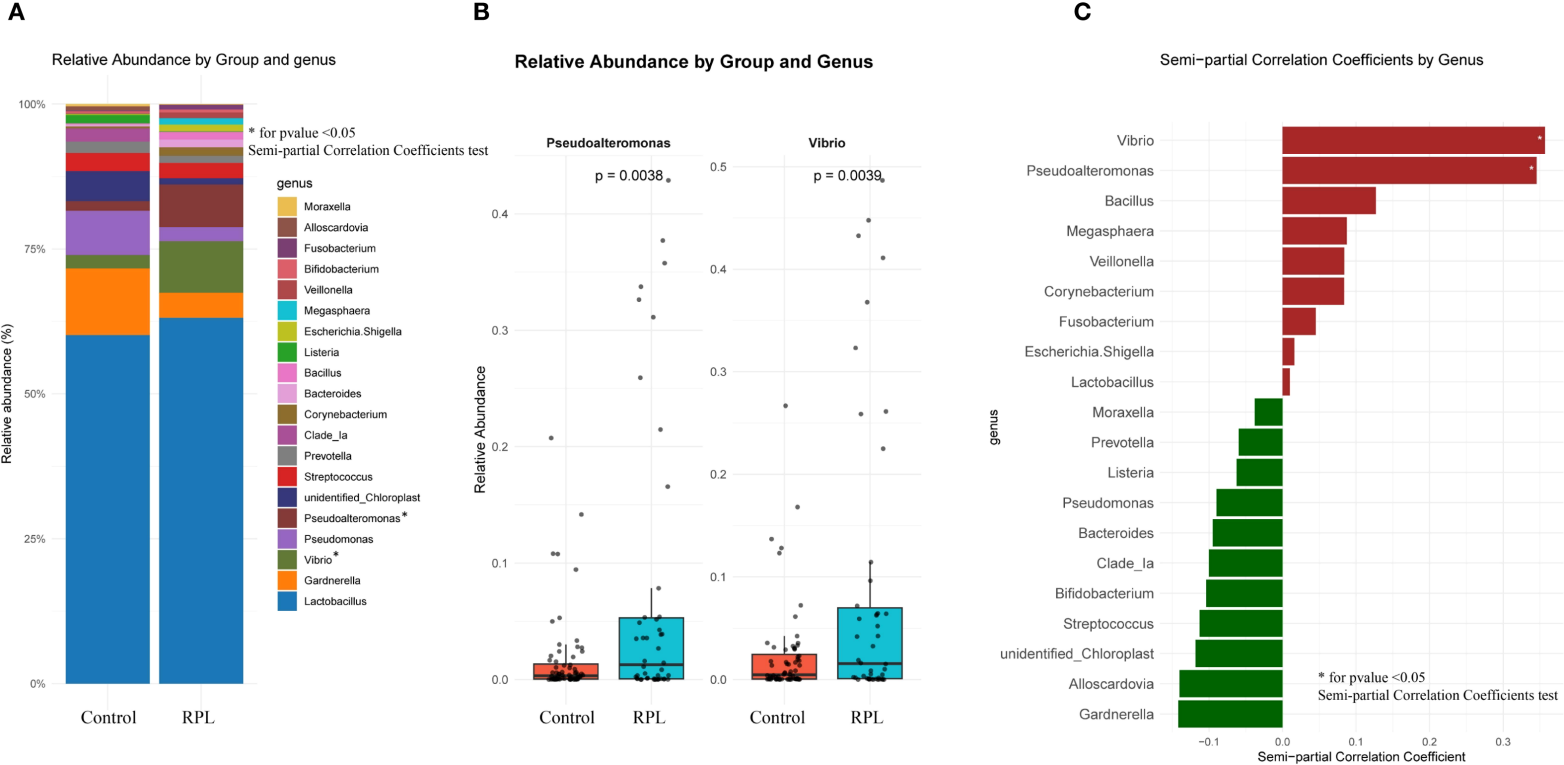
Comparison of microbial composition at the genus levels between the control group and the RPL group. **(A)** Relative abundance of different genera in the control and RPL group. **(B)** Relative abundance of genera with significant differences between the control and RPL group. **(C)** Genera positively and negatively correlated with RPL based on semi-partial correlation coefficients after adjusting for clinical variables using semi-partial correlation analysis.

At the genus level, **Figure 4A** illustrates the relative abundance of various genera in the control and RPL group. **Figure 4B** provides the log2 relative abundance of genera that exhibit significant differences between the two groups. Specifically, *Vibrio* and *Pseudoalteromonas* are significantly more abundant in the RPL group. Finally, **Figure 4C** shows the genera positively and negatively correlated with RPL after adjusting for clinical variables based on semi-partial correlation coefficients. Genera such as *Vibrio* and *Pseudoalteromonas* are positively correlated with RPL, suggesting a potential role in the condition, with p-values < 0.05. The results of the semi-partial correlation analysis at the genus level are presented in **Supplementary Data** – **Supplementary Table S2**.

### Differentially abundant taxa identified by LEfSe analysis

**Figure 5:**
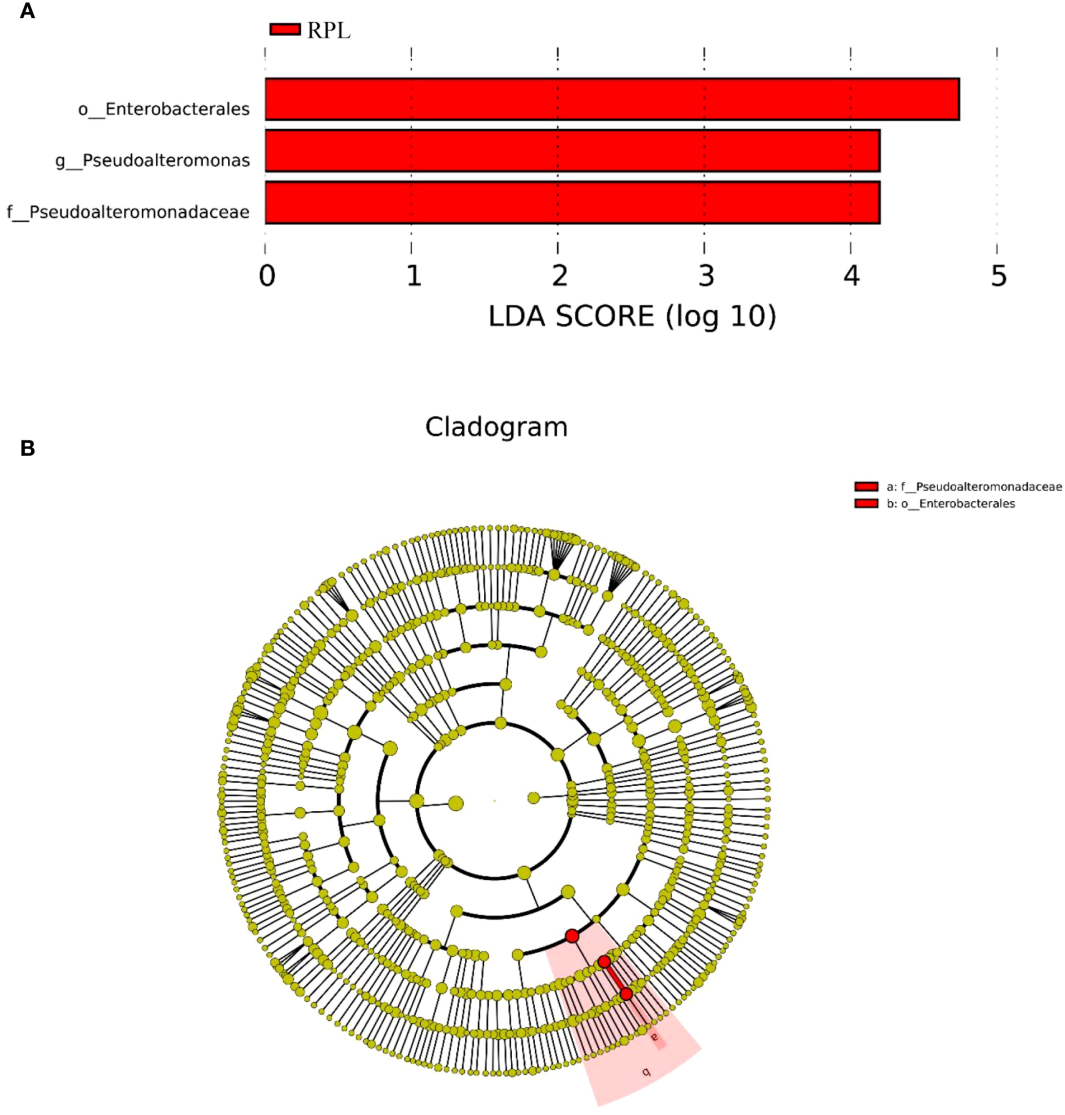
LEfSe analysis of the microbial communities in the RPL group. **(A)** Linear Discriminant Analysis (LDA) scores of bacterial taxa showing significant differences between the RPL group. **(B)** Cladogram representing the taxonomic hierarchy of significantly different bacterial taxa identified by LEfSe analysis.

LEfSe analysis further identified significant differences in the relative abundance of certain genera between the RPL and control groups. **Figure 5A** presents the LDA scores from the LEfSe analysis, indicating the differentially abundant taxa between the RPL and control groups. The taxa significantly enriched in the RPL group are depicted with red bars. The most significantly enriched taxa in the RPL group include *Pseudoalteromonas, Pseudoalteromonadaceae* and *Enterobacterales*. **Figure 5B** provides a cladogram representing the taxonomic hierarchy and relative abundance of the significantly different bacterial taxa. The cladogram visually depicts the relationships among these taxa and their relative abundances in RPL group.

## Discussion

The normal endometrial microbiota can maintain the homeostasis of the endometrial environment, providing favorable conditions for embryo implantation (Kaluanga Bwanga et al., 2023). When the endometrial microbiota becomes dysbiotic, it may disrupt the antimicrobial barrier, cause immune cell imbalances, promote the release of inflammatory factors, and produce abnormal metabolic products, thereby altering the endometrial microenvironment (Moreno et al., 2020; Odendaal et al., 2024). Therefore, studying the interactions between the host and microbiota, as well as the impact of abnormal endometrial microbiota on the local physiology and function of the endometrium, is of great significance.

In this study, we investigated the characteristics of the endometrial microbiota in patients with RPL compared to those with healthy control using 16S rRNA gene sequencing technology. Our analysis revealed no significant differences in alpha diversity indices between the RPL and control groups, indicating that the overall richness and diversity of the microbiota are comparable between the two groups. However, beta diversity analysis showed significant differences, suggesting distinct microbial community structures in RPL patients compared to controls. This finding is consistent with previous studies that have reported altered microbial communities in the reproductive tract of women with adverse reproductive outcomes. Early work suggested that the endometrial microbiota may be associated with implantation and pregnancy outcomes (Franasiak et al., 2016; Moreno et al., 2016). Since then, multiple studies have provided additional evidence that microbial imbalances across the reproductive tract are linked with adverse reproductive outcomes. Chen et al. demonstrated that vaginal microbiome shifts are strongly associated with reproductive health states (Chen et al., 2021), and Peuranpää et al. reported compositional differences in the endometrium and vagina of women with recurrent pregnancy loss, including decreased Lactobacillus crispatus and increased Gardnerella vaginalis (Peuranpää et al., 2022). Takimoto et al. further highlighted the frequent co-occurrence of chronic endometritis and microbial dysbiosis in patients with recurrent implantation failure and RPL (Takimoto et al., 2023). More recently, Odendaal et al. synthesized current evidence on the endometrial microbiota and early pregnancy loss, underscoring the contribution of advanced sequencing technologies (Odendaal et al., 2024), while Gao et al. emphasized the clinical relevance of assessing vaginal and endometrial microbiota in repeated implantation failure and RPL (Gao et al., 2024). The possible explanation for these findings is that specific bacterial taxa in RPL patients may create a less favorable environment for embryo implantation and pregnancy maintenance. For instance, the presence of pathogenic bacteria can induce inflammatory responses, disrupt the endometrial lining, and interfere with normal implantation processes (Benner et al., 2018; Espinós et al., 2021; Negishi et al., 2021). Furthermore, beneficial bacteria, such as Lactobacillus, are known to play a protective role in maintaining a healthy reproductive tract by producing lactic acid and hydrogen peroxide, which inhibit the growth of pathogenic microbes (Moreno et al., 2020; Onyango et al., 2023).

At the phylum level, differences in microbial composition were observed between the control and RPL group. *Firmicutes, Bacteroidota*, and *Proteobacteria* were among the most abundant phyla. Notably, *Cyanobacteria* was more abundant in the control group than in the RPL group. However, after adjusting for confounding factors, *Cyanobacteria* did not show a statistically significant difference between the two groups. At the genus level, the genera *Vibrio* and *Pseudoalteromonas* were found to be significantly more abundant in the RPL group. These genera were positively correlated with RPL, indicating a potential role in the condition. *Vibrio* and *Pseudoalteromonas* are genera commonly found in aquatic environments. *Vibrio* species are well-known for their pathogenic potential in humans, often causing gastrointestinal diseases (Cho et al., 2021). In the context of RPL, their increased abundance could contribute to an inflammatory environment in the endometrium, potentially interfering with embryo implantation and leading to adverse pregnancy outcomes. The pro-inflammatory nature of certain *Vibrio* species might disrupt the delicate immune balance required for successful pregnancy maintenance (Bosi et al., 2017). On the other hand, *Pseudoalteromonas* is primarily known for producing bioactive compounds that can inhibit the growth of other microorganisms (Alviz-Gazitua et al., 2022). These compounds could have dual effects, some might possess antimicrobial properties, while others might disrupt the normal microbial flora balance, leading to dysbiosis. In RPL patients, the increased presence of *Pseudoalteromonas* could indicate a disruption in the protective microbial environment of the endometrium, making it more susceptible to pathogenic invasion or immune dysregulation. Both genera might induce local inflammatory responses, compromising endometrial receptivity and disrupting the implantation process. *Pseudoalteromonas*, through its bioactive compounds, might outcompete beneficial microbiota such as *Lactobacillus*, which are crucial for maintaining a healthy vaginal and endometrial environment (Di Simone et al., 2020). The reduction of beneficial bacteria can lead to an overgrowth of pathogens and an imbalanced immune response (Gholiof et al., 2022). Certain species within these genera might influence local immune responses, altering cytokine profiles and immune cell infiltration in the endometrium, which are critical for successful implantation and pregnancy maintenance. The enrichment of *Vibrio* and *Pseudoalteromonas* in RPL patients highlights the potential role of these genera in the pathogenesis of RPL. Their ability to induce inflammation, disrupt microbial balance, and modulate immune responses could contribute to the adverse reproductive outcomes observed in RPL.

In our study, LEfSe analysis further identified significant differences in the relative abundance of certain genera between the RPL and control groups. Specifically, *Pseudoalteromonas* was significantly more abundant in the RPL group. These findings align with our semi-partial correlation analysis, which showed a significant increase in *Pseudoalteromonas* in the RPL group. *Pseudoalteromonas* is known for producing bioactive compounds that inhibit other microorganisms’ growth. Their increased presence in RPL patients could disrupt the endometrial microbial balance, leading to dysbiosis and creating an inflammatory environment detrimental to embryo implantation and pregnancy maintenance.

These findings underscore the importance of the endometrial microbiota’s composition in reproductive health and highlight the potential role of microbial imbalances in the pathogenesis of RPL. Among the taxa identified, genera such as *Vibrio* and *Pseudoalteromonas*, typically associated with aquatic environments, were enriched in the RPL group. While their detection in low-biomass samples requires cautious interpretation, these genera were absent from the negative controls, supporting that their presence in patient samples is unlikely to be a technical artefact. The biological relevance of these genera remains uncertain and warrants further investigation using metagenomic sequencing and functional studies.

There are several limitations to our study that should be considered. First, the sample size, although sufficient to detect significant differences, is relatively small. This limits the strength and generalizability of the conclusions. Therefore, further studies with more diverse populations are warranted to validate our findings and provide more robust evidence. Second, our study is cross-sectional, which limits the ability to establish causality. The microbial differences observed may either contribute to the development of RPL or result from the condition itself. Longitudinal studies and functional experiments will be necessary to clarify the causal role of microbiota in RPL. Third, while 16S rRNA gene sequencing provides valuable insights into microbial composition, it has inherent limitations, particularly in low-biomass samples such as endometrial fluid. The risk of contamination and background noise in such samples may affect the accuracy of microbial profiling. Although strict aseptic procedures were followed during sample collection and processing, potential biases cannot be entirely ruled out. Future studies should incorporate metagenomic and transcriptomic analyses to provide functional insights into the identified taxa and implement standardized protocols to minimize technical biases.

## Conclusions

In conclusion, our study demonstrates significant differences in the endometrial microbiota between RPL patients and healthy controls, with a notable abundant of *Vibrio* and *Pseudoalteromonas* shift towards the RPL group. These findings underscore the importance of a balanced endometrial microbiota for successful pregnancy and highlight the potential role of microbial dysbiosis in the pathogenesis of RPL.

## Data Availability

All metabolomics data supporting the findings of this study have been deposited in the Figshare repository (https://doi.org/10.6084/m9.figshare.30259045) and are available under a CC BY 4.0 license.
