# Supplementary material for: Analysis of the characteristics of endometrial fluid samples in recurrent pregnancy loss patients based on 16S rRNA gene sequencing technology
Source: Front Cell Infect Microbiol. 2025 Oct 13;15:1646125. doi: 10.3389/fcimb.2025.1646125 (PMC12554706; doi:10.3389/fcimb.2025.1646125)
Supplement: Supplementary file 1 [file DataSheet1.docx]

**Table S1** Semi-partial correlation analysis of phyla with RPL. This table presents the *p*-values, estimates, and direction of correlation for the top 10 Phyla identified. The direction indicates whether the Phylum is positively or negatively correlated with RPL after adjusting for clinical variables such as age, BMI, education level, race, FBG, TC, TG, LDL, and HDL.

| **Phylum** | ***p*-value** | **Estimate** | **Direction** |
| --- | --- | --- | --- |
| Firmicutes | 0.915265289 | -0.010560691 | Negative correlation to RPL |
| Actinobacteriota | 0.11104534 | -0.157183508 | Negative correlation to RPL |
| Proteobacteria | 0.058272485 | 0.186309408 | Positive correlation to RPL |
| Bacteroidota | 0.541700851 | -0.060515775 | Negative correlation to RPL |
| Aenigmarchaeota | 0.649685883 | 0.045059807 | Positive correlation to RPL |
| Cyanobacteria | 0.236715561 | -0.117041178 | Negative correlation to RPL |
| Fusobacteriota | 0.479654257 | 0.07007487 | Positive correlation to RPL |
| Patescibacteria | 0.22464232 | 0.120089545 | Positive correlation to RPL |
| Chloroflexi | 0.865592003 | 0.016799194 | Positive correlation to RPL |
| Campylobacterota | 0.573133262 | 0.055882336 | Positive correlation to RPL |

**Table S2** Semi-partial correlation analysis of genera with RPL. This table provides the *p*-values, estimates, and direction of correlation for the top 20 Genera identified. The direction indicates whether the Genus is positively or negatively correlated with RPL after adjusting for clinical variables.

| **Genus** | ***p*-value** | **Estimate** | **Direction** |
| --- | --- | --- | --- |
| Lactobacillus | 0.920700681 | 0.009930013 | Positive correlation to RPL |
| Gardnerella | 0.153066402 | -0.141800352 | Negative correlation to RPL |
| Listeria | 0.531438248 | -0.06236413 | Negative correlation to RPL |
| Streptococcus | 0.256577596 | -0.112806793 | Negative correlation to RPL |
| Pseudomonas | 0.367011705 | -0.089801275 | Negative correlation to RPL |
| Alloscardovia | 0.157878331 | -0.140175133 | Negative correlation to RPL |
| Bacillus | 0.201296018 | 0.126951274 | Positive correlation to RPL |
| Prevotella | 0.548709008 | -0.059766323 | Negative correlation to RPL |
| Escherichia.Shigella | 0.871936244 | 0.016078665 | Positive correlation to RPL |
| Megasphaera | 0.379713776 | 0.087454993 | Positive correlation to RPL |
| unidentified_Chloroplast | 0.233303864 | -0.118472287 | Negative correlation to RPL |
| Vibrio | 0.000214684 | 0.356944841 | Positive correlation to RPL |
| Corynebacterium | 0.400123208 | 0.083782326 | Positive correlation to RPL |
| Pseudoalteromonas | 0.000351309 | 0.345483125 | Positive correlation to RPL |
| Fusobacterium | 0.651498121 | 0.045032543 | Positive correlation to RPL |
| Veillonella | 0.39946862 | 0.08389836 | Positive correlation to RPL |
| Bifidobacterium | 0.296524441 | -0.103849036 | Negative correlation to RPL |
| Moraxella | 0.703880177 | -0.037900255 | Negative correlation to RPL |
| Clade_Ia | 0.314516873 | -0.100075668 | Negative correlation to RPL |
| Bacteroides | 0.341021297 | -0.094763225 | Negative correlation to RPL |
